# Perspectives on the therapeutic potential of MDMA: A nation-wide exploratory survey among substance users

**DOI:** 10.3389/fpsyt.2023.1096298

**Published:** 2023-04-14

**Authors:** Jennifer L. Jones

**Affiliations:** Department of Psychiatry and Behavioral Sciences, Medical University of South Carolina, Charleston, SC, United States

**Keywords:** MDMA, PTSD, substance and alcohol use, treatment, race and ethnicity

## Abstract

**Background:**

Alcohol and other substance use disorders are commonly associated with post-traumatic stress disorder (PTSD), and the presence of these comorbidities is associated with worse treatment outcomes. Additionally, disparities in substance and PTSD prevalence have been associated with minority races and ethnicities, and minorities have been shown to be less likely to engage in treatment. Psychedelic-assisted treatments, including 3,4-methylenedioxymethamphetamine (MDMA), have shown preliminary trans-diagnostic effectiveness, however it is unknown how individuals with substance use disorders view the therapeutic potential of MDMA therapy. Previous studies have also shown that minority races and ethnicities are under-represented in the MDMA trials, leading to concerns about inequitable access to clinical treatment.

**Methods:**

To explore demographic characteristics related to patient-level perspectives on the therapeutic potential of MDMA-assisted therapy, this study describes data from a nationwide, cross-sectional survey of 918 individuals self-reporting criteria consistent with alcohol or substance use disorders.

**Results:**

Overall, a majority of individuals reported support for medical research of MDMA (68.1%), belief that MDMA-assisted therapy might be a useful treatment (70.1%), and willingness to try MDMA-assisted therapy if it were determined to be an appropriate treatment for them (58.8%). No race or ethnicity differences were found in support for further research or belief in effectiveness, however there were small disparities in terms of willingness to try MDMA-assisted therapy and concerns related to use of this treatment approach.

**Conclusion:**

These results provide insights and future directions as the field of psychedelic-assisted therapy seeks to provide equitable access to clinical care and to diversify research participation.

## Introduction

Substance use disorders (including alcohol and tobacco) are highly prevalent globally, and commonly occur in the context of other mental health disorders, including post-traumatic stress disorder (PTSD). Substance use varies as a function of legalization status and cultural norms, and prevalence rates of PTSD vary as a function of population exposure to war, regional traumas, and intra-familial violence as well as cultural values and other influences. A reciprocal relationship between these comorbidities has been noted. Individuals with substance use disorders are more likely to experience traumatic events and to develop PTSD, and individuals with primary PTSD are more likely to develop substance use disorders (hypothetically to self-medicate PTSD symptoms) (1). Previous work has shown individuals with a substance use disorder are 6.5 times more likely to have PTSD (2). Additionally, studies of trauma centers have suggested that between 62 and 79% of individuals with elevated PTSD symptoms have 1 or more concurrent substance use disorders (3). Furthermore, individuals with these comorbid disorders are more likely to experience symptoms that are refractory to treatment (4–6).

The prevalence of PTSD has been noted in some studies to vary as a function of race and ethnicity. Among Vietnam Veterans, individuals identifying as American Indian have been found to be more likely than those identifying as (non-Hispanic) White to report symptoms consistent with PTSD and were shown to have elevated prevalence of lifetime PTSD (7). Other studies of Vietnam Veterans found that individuals identifying as African American or Hispanic were found to be at higher risk of developing PTSD compared to individuals identifying as non-Hispanic White (8). A later study of individuals living nearby during the terrorist attack in New York City on September 11, 2001 also showed that Hispanic individuals were more likely to have symptoms of PTSD (9). Rates of PTSD among American Indian/Alaskan Natives have also been shown to be elevated compared to the general population (10). More recently, lifetime prevalence of PTSD has been estimated to be 8.7% among Blacks, 7.0% among Hispanics, 7.4% among Whites and 4.0% among Asians in a structured diagnostic interview of the U.S. general population (11). Additionally, an increasing body of research supports the development of PTSD symptoms in response to race-based trauma, previously defined as a cumulative traumatic effect of racism on an individual (12).

Notably, rates of both substance use disorder and PTSD treatment have been shown to be significantly lower in all minority race and ethnicities compared to individuals self-reporting as White (11). Compared to White individuals, Blacks and Hispanics have been shown to have significantly lower substance use disorder treatment completion rates (13, 14). Other studies have shown significant differences in treatment location, with Black and Hispanic individuals less likely to receive treatment at a doctor’s office compared to Whites, and Black individuals are more likely than Whites to receive treatment through the criminal justice system (15). Additionally, previous work has shown that Black individuals undergoing treatment had worse outcomes compared to White individuals, while Latino individuals had improved treatment outcomes compared Whites and Blacks. (16). Similarly, studies of individuals with PTSD have shown reduced treatment engagement among Blacks, Hispanics, and Asians compared to Whites (11). Furthermore, among individuals seeking integrated treatment for comorbid substance use disorders and PTSD, studies of veterans have shown reduced rates of treatment response in African Americans compared to Whites (17).

Despite the high prevalence of these comorbidities, there are no pharmacologic treatments which have been shown to be effective in improving symptoms of both types of disorders. Within the United States, there are medications approved for alcohol use disorder (disulfuram, naltrexone, and acamprosate) and three medications approved for opioid use disorder treatment (methadone, buprenorphine, and naltrexone). There are currently no approved pharmacotherapies for cocaine, amphetamine, cannabis, or benzodiazepine use disorders. Sertraline and paroxetine, the only two approved medications for PTSD, have shown only modest effects in PTSD (18). Similarly, while multiple behavioral therapies for combined PTSD and substance use disorders have been explored (i.e., cognitive behavioral therapy, mindfulness based therapies, and prolonged exposure therapy), treatment outcomes remain limited (19).

Compounds with psychedelic properties have been explored for their trans-diagnostic effects in the treatment of mental health disorders. Psilocybin and ketamine are among the more well studied compounds with psychedelic effects, and both have shown preliminary effectiveness in multiple types of substance use disorders (including alcohol and tobacco) as well as other mental health disorders including major depressive disorder, PTSD, and anxiety-spectrum disorders (20–28). Further, 3,4-methylenedioxymethamphetamine (MDMA), a compound with non-classical psychedelic effects received breakthrough therapy status designation from the United States Food and Drug Administration in 2017, indicative of its potential therapeutic advantage over existing treatment options. To date, MDMA has been studied in conjunction with therapy in more than 11 Phase 2 and two Phase 3 trials for the treatment of PTSD. Results from the initial Phase 3 trial showed that MDMA-assisted therapy produced a substantial decrease in symptoms associated with severe PTSD (29). In this study, after completing three sessions of MDMA-assisted therapy, 67% of the participants in the MDMA group no longer met diagnostic criteria for PTSD (a certain number of criteria from each of the different PTSD symptom categories), compared to 32% in the placebo-assisted therapy group. Further, 33% of the MDMA-assisted therapy group met criteria for PTSD remission (defined as loss of diagnosis and a CAPS-5 score of less than or equal to 11), compared to 5% in the placebo-assisted therapy group. In this Phase 3 study, subjects were permitted to have mild alcohol or cannabis use disorders, or moderate alcohol or cannabis use disorders (if in early remission); other substance use disorders were excluded. A subsequent secondary analysis of the Phase 3 MDMA-assisted therapy trial data explored changes in assessments of alcohol and drug use severity using the Alcohol and Drug Use Disorders Identification Tests (AUDIT and DUDIT assessments, respectively). The authors found that MDMA-assisted therapy was associated with greater reductions in alcohol use severity, but not drug use severity and did not find a correlation between changes in PTSD severity and AUDIT score reduction (30). Other studies of MDMA-assisted therapy for the treatment of alcohol use disorder have shown preliminary effectiveness in reducing alcohol consumption (31).

Concerningly, demographic analyses of MDMA and other psychedelic-assisted therapy studies have shown that minority races and ethnicities are significantly underrepresented in psychedelic clinical trials. According to the 2021 United States Census, 13.1% of the population identified as Black or American India, 1.3% as American Indian or Alaskan Native, 6.1% as Asian, 0.3% as Native Hawaiian or Pacific Islander, 2.9% as Multi-Racial, and 18.9% as Hispanic or Latino (32). Overall, 40.7% of the population reported being of a race or ethnicity that was not White alone/not Hispanic or Latino. However, a 2018 systematic review of 18 previous psychedelic studies showed that only 17.4% of the aggregated sample identified as Black, Indigenous, or a Person of Color (33). Additionally, in the 2019 analysis of the six Phase 2 MDMA-assisted therapy trials, only 12.4% of participants self-reported as identifying as Black, Indigenous, or a Person of Color (34). While sub-analyses have supported that psychedelics and MDMA-assisted therapy is equally safe and efficacious in these populations (including in its ability to promote healing from racial trauma), other studies have suggested decreased interest or willingness to participate in psychedelic-assisted therapy (35, 36).

Minority under-representation in clinical trials may occur from a variety of psychosocial concerns. Experienced or perceived barriers to participation include mistrust related to historical abuses of minorities in medical care and research participation (including the racial and ethnic criminal injustices related to the United States “War on Drugs” in the early 1970’s), as well as ineffective communication and cultural messaging in the recruitment process, lack of appropriate logistical support such as childcare and transportation to facilitate participation, and overt and subtle racism (37, 38).

Given the preliminary trans-diagnostic effects of MDMA and other psychedelics in previous trials, this study sought to explore patient-level opinions and beliefs on the research and clinical potential of psychedelic-assisted therapies among substance users. Level of support for further research into MDMA-assisted therapy was assessed, as well as beliefs about whether MDMA-assisted therapy might be a beneficial treatment. Additionally, the study assessed subject willingness to try MDMA-assisted therapy if it was deemed an appropriate treatment option for them and whether they had any concerns about trying the treatments. In addition to aggregate data about these patient-level perspectives, this study explored differences in opinions and beliefs as a function of race and ethnicity.

## Methods

### Study design and ethical review

A cross-sectional survey study was designed and administered to assess substance users’ perspectives on the use of several psychedelics including MDMA. This study was declared exempt from review by the Medical University of South Carolina’s Institutional Review Board (IRB). In order to protect anonymity, written informed consent was not obtained. However, the pre-screening portion of the survey described the study and its purpose, and informed potential participants that submitting their responses constituted consent. Prior to completion of the main survey, potential participants were required to first complete a pre-screener questionnaire to verify inclusion criteria. Internet Protocol (IP) constraints were set up to prevent participants from taking the survey more than once.

### Recruitment

Recruitment was conducted through advertisements placed on the internet (i.e., Craigslist). Online advertisements invited individuals who “struggle with alcohol or drug use” to participate in a short survey about the use of psychedelic substances in addictions treatment. Both pre-screening and the main survey were conducted using the secure web database, REDCap.

### Inclusion criteria

To be eligible for participation in the main survey, participants must have self-reported giving consent to participate in the study, being over the age of 18 and use of a substance of abuse at least once in the past month. Additionally, participants must have self-reported at least two of the following: (1) wanting to cut back on or stop a substance of abuse, (2) previously having been in treatment for alcohol or drug use, or (3) currently being in treatment for alcohol or drug use. Pre-screening questions are shown in Supplementary Figure 1.

### Exclusion criteria

Potential participants were excluded if they (1) did not self-report at least 2 criteria for a substance use disorder, (2) did not report using a substance of abuse at least once per month, (3) denied ever wanting to cut back on or stop using a substance of abuse, or (4) did not give their consent to participate in the study.

### Screening and informed consent

Initial screening eligibility using the inclusion and exclusion criteria was conducted using a pre-screener questionnaire hosted *via* REDCap. Participants were informed that participation in the study was voluntary, and they could discontinue at any time. Participants were provided with an overview of the study procedures in advance on the pre-screening questionnaire.

### Assessment procedures

Following completion of informed consent, and provided that all inclusion and exclusion criteria were satisfied, eligible participants proceeded to take the full survey. The survey took approximately 20 min to complete. The survey was anonymous, but participants were asked basic questions about themselves such as their age, gender, race, and use of alcohol and various substances. Participants were supplied with the following information about MDMA-assisted therapy: “In 2016, the FDA approved MDMA (also known as ecstasy) for Phase 3 clinical trials as a treatment for post-traumatic stress disorder (PTSD), which is a common disorder that occurs with addictions. These studies are one of the final steps before possible approval as a prescription drug. One previous study showed that with 3 doses of MDMA administered under a psychiatrist’s guidance, the patients reported a 56% decrease in severity of PTSD symptoms on average. At the end of the study, 2/3 of the study participants (66%) no longer met the criteria for having PTSD. Improvements lasted more than a year after therapy.” Participants were subsequently asked (1) “Based on these findings and what you may have known previously, do you support or oppose similar medical trials with MDMA being conducted in the future,” (2) “do you think that MDMA could or could not be a beneficial treatment for people suffering from PTSD,” and (3) if MDMA is proven to be safe and effective for treatment after further trials, would you or would you not try this treatment if it was appropriate for you?”

### Participant compensation

Participants who completed the full survey were eligible to be compensated for their time with a $15 Amazon gift card. This gift card would be sent to their email address. If the participant did not wish to provide their email address, they could still take the survey, but they would not be able to receive compensation.

### Data analytic procedure

All data from this survey was collected and managed using the secure REDCap (Research Electronic Data Capture) database. Data was analyzed using the SPSS statistical software platform. Baseline demographic characteristics were collected from all participants and descriptive statistics for the sample population were analyzed. Descriptive statistics were analyzed with regard to overall sample with regard to regard to (1) level of support for further clinical trial research into MDMA assisted therapy, (2) subject belief that MDMA might be a beneficial treatment, and (3) subject willingness to try MDMA-assisted therapy. Additionally, Kruskal-Wallis H tests were used to evaluate potential differences between race and ethnicity group differences with regard to these three variables. Descriptive statistics were also analyzed with regard to proportion of the total sample expressing different potential concerns related to use of psychedelics.

## Results

### Demographic profile of respondents

Of the 935 individuals that initiated the survey, 918 (98.2%) individuals completed responses to the full survey and were included in the analysis. The demographic profile of respondents was generally diverse. Of the respondents that completed the full survey, a majority of individuals (70.9%) self-identified as male, while 28.2% of respondents identified as female, 0.4% as transgender or non-binary, and 0.4% as other or responded that they preferred not to gender identify. Survey respondents self-identified as being of one or more of the following race or ethnicity categories: American Indian or Alaska Native (4.4%), Asian (2.4%), Black or African American (20.6%), Hispanic or Latino (13.6), Native Hawaiian or Other Pacific Islander (0.7%), White (56.2%), Multi-racial (1.7%) or Other/Preferred Not to Answer (0.4%).

### Overall level of support for MDMA therapy

A majority of individuals reported either supporting or strongly supporting medical research of MDMA (68.1%). Furthermore, a majority reported either strongly or very strongly believing that MDMA could be useful for the treatment of mental health disorders such as addiction and PTSD (70.1%). Similarly, if MDMA was proven to be a safe and effective treatment of a disorder that they suffered from, a majority stated that they would personally be willing to therapeutically use it (58.8%).

### Racial and ethnic differences

**Figure 1:**
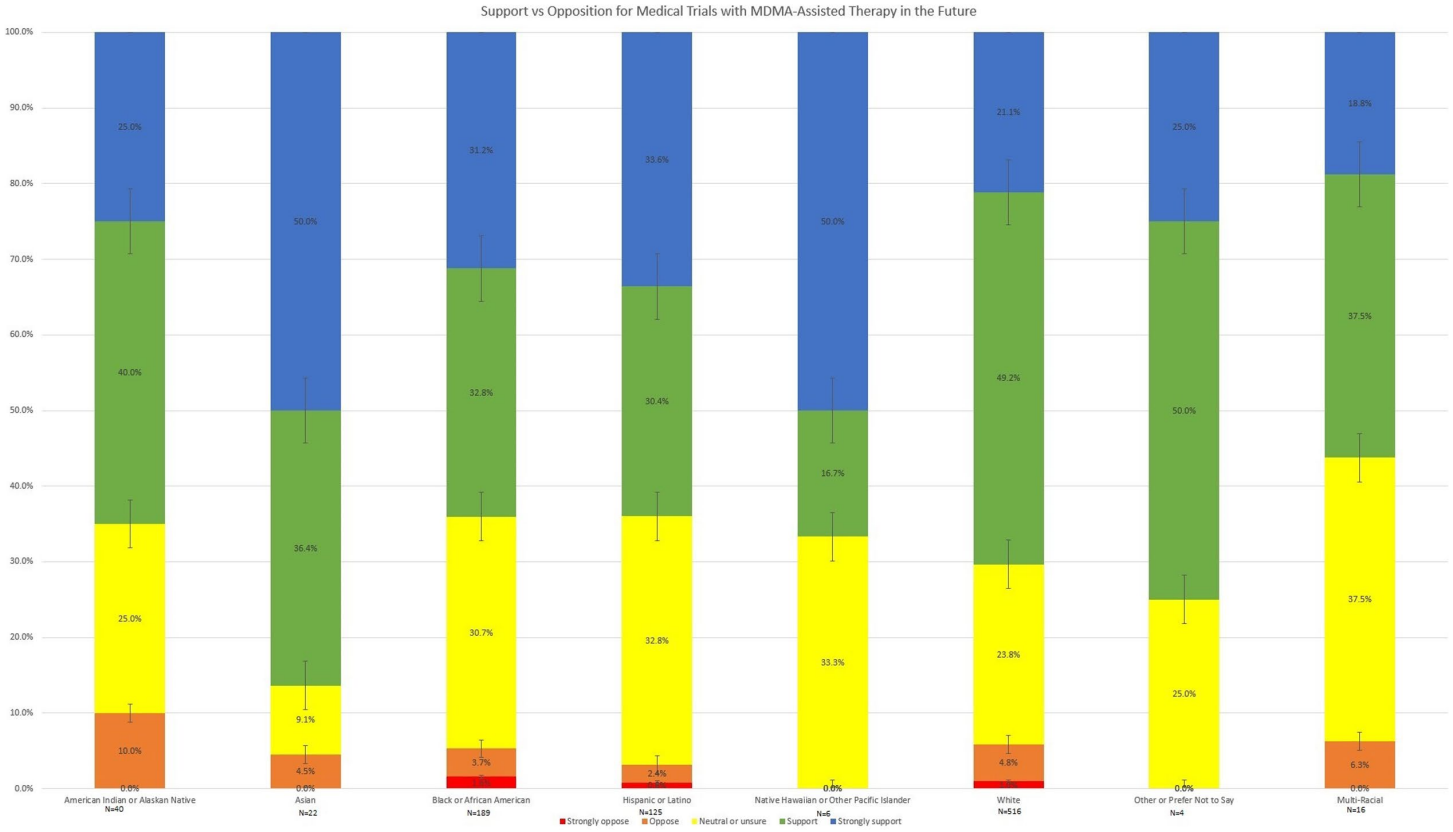
Support vs opposition for medical trials with MDMA-assisted therapy in the future.

**Figure 2:**
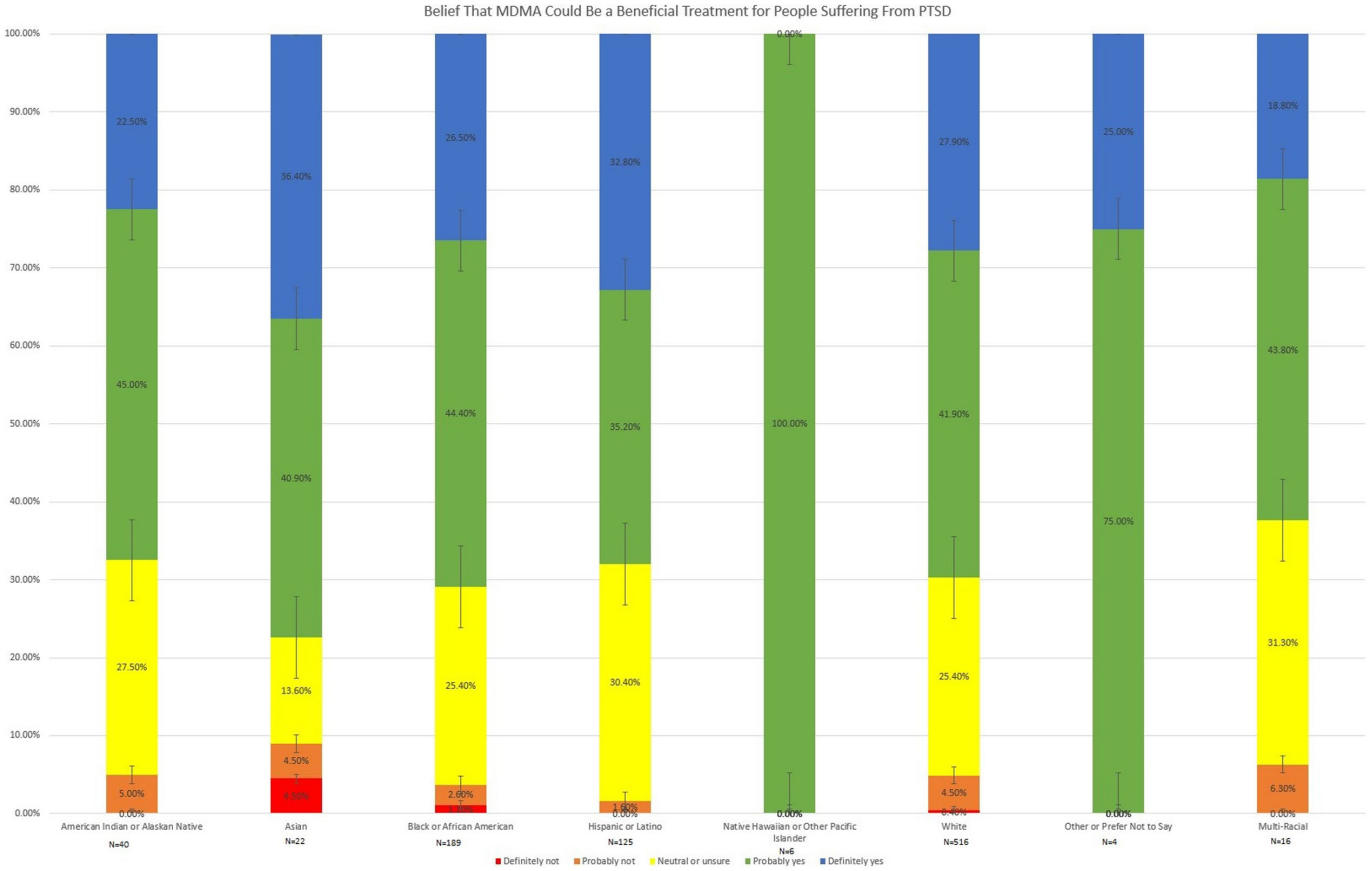
Belief that MDMA-assisted therapy could be a beneficial treatment for people suffering from PTSD.

**Figure 3:**
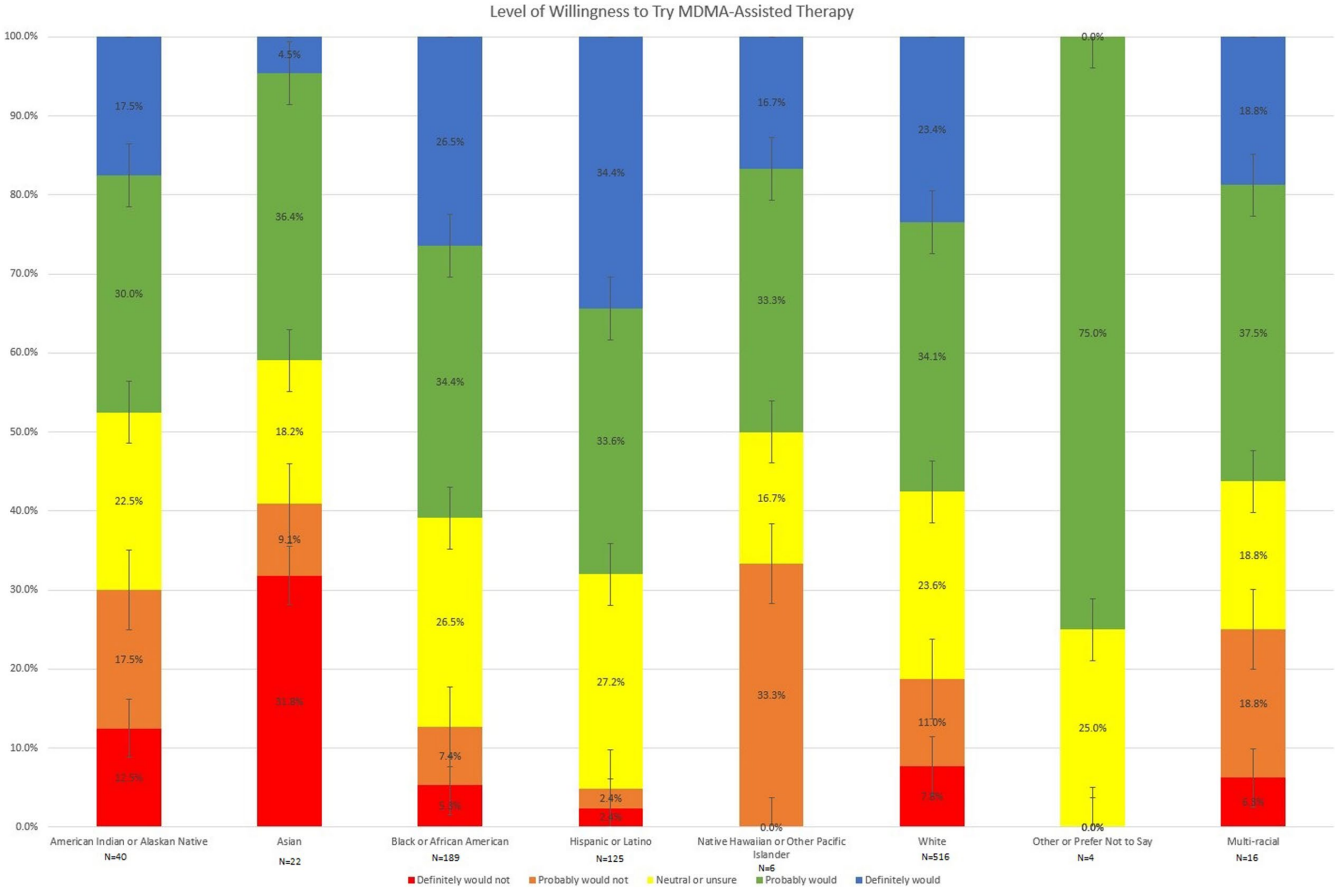
Level of willingness to try MDMA-assisted therapy.

Descriptive analysis of level of support for MDMA trial research, belief that MDMA-assisted therapy could be a beneficial treatment for PTSD, and willingness to try MDMA-assisted therapy (if it were appropriate for them) by race and ethnicity are shown in Figures 1–3. Potential race and ethnicity group differences with regard to level of support for further clinical trial research into MDMA assisted therapy was assessed using a Kruskal-Wallis H test. Distributions of level of support were not similar for all groups, as assessed by visual inspection of a boxplot. The mean rank of level of support scores was not statistically significantly different between groups, *χ*^2^(7) = 9.198, *p* = 0.239.

Similarly, race and ethnicity differences with regard to subject belief that MDMA might be a beneficial treatment for individuals suffering from PTSD were analyzed using a Kruskal-Wallis H test. Distributions of level of belief in benefit were not similar for all groups, as assessed by visual inspection of a boxplot. The mean rank of level of support scores was not statistically significantly different between groups, *χ*^2^(7) = 2.763, *p* = 0.906.

Finally, group differences with regard to subject willingness to try MDMA-assisted therapy were analyzed using a Kruskal-Wallis H test and are shown in Figure 3. Distributions of level of willingness to try MDMA-assisted therapy were not similar for all groups, as assessed by visual inspection of a boxplot. A between group difference in subject willingness to try MDMA-assisted therapy was found, *χ*^2^(7) = 24.699, *p* < 0.001. Pairwise comparisons were performed using Dunn’s procedure with a Bonferroni correction for multiple comparisons. As shown in Figure 3, Asians to were less likely to report willingness to try MDMA-assisted therapy compared to Whites (*p* = 0.029) or Hispanics or Latinos (*p* = 0.002). American Indians and Alaskan Natives were also reported decreased willingness to try MDMA-assisted compared to Hispanics or Latinos (*p* = 0.041). Among Hispanic/Latino respondents, 68.0% reported that they definitely or probably would be willing to try MDMA-assisted therapy, while only 57.5% of White respondents, 47.5% of American Indian and Alaskan Natives, and 40.9% of Asians reported definite or probable willingness to try this treatment. No other significant pairwise comparisons emerged.

### Concerns

Despite overall high levels of support for MDMA research trials, belief that MDMA could be a beneficial treatment, and willingness to try the treatment if it was an appropriate treatment for their condition, a majority (96.2%) of individuals also expressed concerns about psychedelic-assisted therapies. Concerns endorsed included fear of a bad trip (31.8%), fear that it would change [them] (35.4%), fear that it would cause [them] to go crazy (39.9%), fear that [they] would feel guilt during the experience (32.8%), fear of losing [their] sense of self (31.5%), fear that it would affect employment (26.7%), fear that family, friends or neighbors would find out (27.3%), and fear that they would no longer enjoy using substances (35.4%). Survey respondents were also queried about non-listed concerns, and 2.7% indicated that they had additional concerns. Of the 7 concerns that were submitted, 4 related to concerns about becoming addicted to a new substance, 1 expressed concern that they would say something they would regret, 1 was concerned about potential side effects, and 1 expressed concern that because they had previously done psychedelics, that they would not gain benefit from their therapeutic use. Survey respondents reported a mean number of 2.94 concerns, with a range of 0–10 of these concerns. Total number of concerns by race and ethnicity is shown in Table 1. Only 4.8% of respondents denied having any concerns about use of psychedelic-assisted therapies, and the independent binomial proportions of respondents with no concerns were not statistically significantly different between race/ethnicities (*p* > 0.05). A Kruskal-Wallis H test was subsequently conducted to determine if there were differences in the mean number of concerns reported between the racial and ethnicity groups. Values are mean ranks unless otherwise stated. Distributions of number of concerns were not similar for all groups, as assessed by visual inspection of a boxplot. The mean ranks of number of concerns were statistically significantly different between groups, *χ*^2^(3) = 14.468, *p* = 0.002. Pairwise comparisons were performed using Dunn’s procedure with a Bonferroni correction for multiple comparisons. Asians reported a fewer number of concerns than Whites (*p* = 0.018) or than African American (*p* = 0.004). American Indians/Alaskan Natives also reported a fewer number of concerns than Whites (*p* = 0.019) or African Americans (*p* = 0.003).

**Table 1:** Total number of concerns by race and ethnicity.

Total # Concerns	AIAN	Asian	Black /AA	Hisp/Latx	Haw/PI	White	Other /Prefer Not to Say	Multi-racial	Total
0	7.5%		2.1%	5.6%		5.6%		6.3%	4.8%
1	17.5%	40.9%	5.8%	7.2%	16.7%	4.8%		6.3%	6.9%
2	30.0%	22.7%	10.6%	6.4%	33.3%	15.5%	25.0%	37.5%	14.6%
3	30.0%	22.7%	59.8%	72.8%	50.0%	51.9%	50.0%	18.8%	54.1%
4	12.5%	13.6%	10.6%	4.8%		11.8%		18.8%	10.7%
5	2.5%		4.8%			6.4%	25.0%		4.8%
6			1.1%	2.4%		1.9%		6.3%	1.7%
7			4.2%			1.0%		6.3%	1.5%
8			0.5%			0.4%			0.3%
9			0.5%	0.8%					0.2%
10						0.6%			0.3%
Mean	2.30	2.09	3.17	2.79	2.33	2.98	3.25	2.94	2.94

AIAN, American Indian or Alaskan Native; AA, African American; Hisp/Latx, Hispanic or Latinx.

## Discussion

Engagement in clinical treatments for PTSD and substance use disorders have been shown to be significantly lower in minority race and ethnicities compared to individuals self-reporting as White, and individuals with alcohol or substance use disorders are more likely to experience symptoms that are refractory to PTSD treatment. MDMA and other psychedelic-assisted therapies represent an exciting new trans-diagnostic treatment approach, however minority races and ethnicities have been significantly under-represented in previous research trials. These disparities in psychedelic research trials have further prompted concern that clinical adoption of these treatments may also be inequitable, although reasons behind the reduced research representation remain largely unstudied. Importantly, no previous studies have directly assessed patient-level perspectives on psychedelic-assisted therapies as a function of race and ethnicity.

The results of this study indicate that an overall majority of individuals self-reporting criteria for substance use disorders (including alcohol) support continued clinical trial research of MDMA-assisted therapy, with no between-group differences in level of support found between the races and ethnicities. Similarly, an overall majority reported either strongly or very strongly believing that MDMA could be useful for the treatment of mental health disorders such as addiction and PTSD, and no between-group differences were found with regard to this belief.

Most germane to clinical treatment adoption, a majority of individuals with substance use disorders reported that they would be willing to try MDMA-assisted therapy if it were determined to be an appropriate treatment for their condition. Pairwise comparisons reported small between group differences. Asians to were less likely to report willingness to try MDMA-assisted therapy compared to Whites, while Hispanics and Latinos were more likely to report willingness to try MDMA-assisted therapies compared to American Indians and Alaskan Natives or Asians. Previous work has suggested that the disproportionate illicit use of MDMA among Asian Americans may relate to feelings of acculturation stress and a desire for social connectedness (39). Willingness to try a treatment modality may be influenced by both cultural and individual perceptions about the potential risks and benefits of the treatment, as well as prior experience with MDMA, which was not assessed in this study. Interestingly, there were no between group differences regarding the belief that MDMA-assisted therapy could be a beneficial treatment in general, which suggests that reduced willingness to try this treatment might be related either to greater assessment of potential risks or reduced belief that it would be of personal benefit. Future research studies and clinical work should examine not only levels of race and ethnicity participation, but concerns related to potential risks or harms that might be addressable with specific education or outreach efforts.

One of the strengths of this survey is the relatively large sample size (918 unique survey completers) which generally represented a diverse demographic profile. Compared to national averages taken from US Census Data, there were higher proportions of all minority races except Asian Americans (4.4% of survey respondents, but 6.1% of the US population); Hispanics/Latinos were also slightly underrepresented compared to national averages (18.9% nationally but only 13.6% of survey respondents) (24). Several important study limitations are to be noted. First, participants self-selected to participate in this online survey, potentially introducing selection bias. Additionally, participants were only eligible for participation if they self-reported 2 criteria consistent with a substance use disorder (including alcohol) as per Supplementary Figure 1, these questions are not validated for formal diagnostic assessment. Additionally, PTSD and other psychiatric symptomatology was not assessed. Given that there has been more limited research into MDMA-assisted therapy for substance use disorders compared to PTSD, this may have influenced subject perception of treatment efficacy or willingness to try MDMA-assisted therapy. Further, while this study evaluated potential concerns specifically about the use of psychedelics, it did not survey general concerns related to research engagement, or what criteria subjects felt was essential for a treatment to “be proven safe and effective.” Further level of prior knowledge about the treatment (which could vary by race/ethnicity) was not characterized.

Importantly, this study was not designed to assess the efficacy of MDMA-assisted therapy in the treatment of substance use disorders (either singularly or co-occurring with PTSD), nor was this exploratory study designed to provide conclusive results about patient-level opinions on MDMA-assisted therapy. However, this study does provide a valuable descriptive analysis of substance users’ belief in the therapeutic potential of MDMA-assisted therapy and their willingness to use it if they were found to be a clinically appropriate treatment for them. Additionally, this study demonstrated that a majority of individuals had some concerns about psychedelic-assisted therapies, which provide direction into further research, public education and counseling strategies related to these treatments.

MDMA and other psychedelic-assisted therapies offer enormous promise in the treatment of refractory and comorbid mental health disorders. However, much work remains ahead. As MDMA and other psychedelics advance toward becoming clinical treatments, improving research diversity and ensuring equitable access to care are of paramount importance.

## Data availability statement

The original contributions presented in the study are included in the article/Supplementary material, further inquiries can be directed to the corresponding author.

## Ethics statement

The studies involving human participants were reviewed and approved by Institutional Review Board of the Medical University of South Carolina. The patients/participants provided their written informed consent to participate in this study.

## Author contributions

JJ designed and implemented the study design, searched for the references, and drafted the manuscript including figures and tables.

## Funding

This research was supported by the National Institute on Drug Abuse (K12DA031794).

## Conflict of interest

The author declares that the research was conducted in the absence of any commercial or financial relationships that could be construed as a potential conflict of interest.

## Publisher’s note

All claims expressed in this article are solely those of the authors and do not necessarily represent those of their affiliated organizations, or those of the publisher, the editors and the reviewers. Any product that may be evaluated in this article, or claim that may be made by its manufacturer, is not guaranteed or endorsed by the publisher.

## Supplementary material

The Supplementary material for this article can be found online at: https://www.frontiersin.org/articles/10.3389/fpsyt.2023.1096298/full#supplementary-material

## References

[1] Gielen N Havermans R Tekelenburg M Jansen A. Prevalence of post-traumatic stress disorder among patients with substance use disorder: it is higher than clinicians think it is. Eur J Psychotraumatol. (2012) 3:17734. doi: 10.3402/ejpt.v3i0.17734, PMID: 22893849 PMC3415609

[2] Mills K Teeson M Ross J Peters L. Trauma, PTSD, and substance use disorders: findings from the Australian National Survey of mental health and well-being. Am J Psychiatry. (2006) 163:652–8. doi: 10.1176/ajp.2006.163.4.652, PMID: 16585440

[3] Nguyen J Whiteside LK Bulger EM Veach L Moloney K Russo J. Post-traumatic stress disorder (PTSD) symptoms and alcohol and drug use comorbidity at 25 US level I trauma centers. Trauma Surg Acute Care Open. (2022) 7:e000913. doi: 10.1136/tsaco-2022-000913, PMID: 35979039 PMC9358953

[4] Bedard-Gilligan M Garcia N Zoellner LA Feeny NC. Alcohol, cannabis, and other drug use: engagement and outcome in PTSD treatment. Psychol Addict Behav. (2018) 32:277–88. doi: 10.1037/adb0000355, PMID: 29595297 PMC9377391

[5] Norman SB Haller M Hamblen JL Southwick SM Pietrzak RH. The burden of co-occurring alcohol use disorder and PTSD in U.S. military veterans: comorbidities, functioning, and suicidality. Psychol Addict Behav. (2018) 32:224–9. doi: 10.1037/adb0000348, PMID: 29553778

[6] Tripp JC Jones JL Back SE Norman SB. Dealing with complexity and comorbidity: comorbid PTSD and substance use disorders. Curr Treat Options Psychiatry. (2019) 6:188–97. doi: 10.1007/s40501-019-00176-w

[7] Beals J Manson SM Shore JH Friedman M Ashcraft M Fairbank JA. The prevalence of posttraumatic stress disorder among American Indian Vietnam veterans: disparities and context. J Traumat Stress. (2002) 15:89–97. doi: 10.1023/A:1014894506325, PMID: 12013069

[8] Kulka RA Schlenger WE Fairbank JA Hough RL Jordan BK Marmar CR. Trauma and the Vietnam war generation. New York: Brunner/Mazel (1990).

[9] Galea S Ahern J Resnick H Kilpatrick D Bucuvalas M Gold J. Psychological sequelae of the September 11 terrorist attacks in New York City. N Engl J Med. (2002) 346:982–7. doi: 10.1056/NEJMsa013404, PMID: 11919308

[10] Bassett D Buchwald D Manson S. Posttraumatic stress disorder and symptoms among American Indians and Alaska natives: a review of the literature. Soc Psychiatry Psychiatr Epidemiol. (2014) 49:417–33. doi: 10.1007/s00127-013-0759-y, PMID: 24022752 PMC3875613

[11] Roberts AL Gilman SE Breslau J Breslau N Koenen KC. Race/ethnic differences in exposure to traumatic events, development of post-traumatic stress disorder, and treatment-seeking for post-traumatic stress disorder in the United States. Psychol Med. (2011) 41:71–83. doi: 10.1017/S0033291710000401, PMID: 20346193 PMC3097040

[12] Williams MT Haeny A Holmes S. Posttraumatic stress disorder and racial trauma. PTSD Res Q. (2021) 32:1–9.

[13] Mennis J Stahler GJ. Racial and ethnic disparities in outpatient substance use disorder treatment episode completion for different substances. J Subst Abus Treat. (2016) 63:25–33. doi: 10.1016/j.jsat.2015.12.007, PMID: 26818489

[14] Mennis J Stahler GJ Abou El Magd S Baron DA. How long does it take to complete outpatient substance use disorder treatment? Disparities among blacks, Hispanics, and whites in the US. Addict Behav. (2019) 93:158–65. doi: 10.1016/j.addbeh.2019.01.041, PMID: 30711669

[15] Archibald ME Behrman P Yakoby J. Racial-ethnic disparities across substance use disorder treatment settings: sources of treatment insurance, socioeconomic correlates and clinical features. J Ethn Subst Abus. (2022)21:1–25. doi: 10.1080/15332640.2022.2129537, PMID: 36208872

[16] Sahker E Pro G Sakata M Furukawa TA. Substance use improvement depends on race/ethnicity: outpatient treatment disparities observed in a large US national sample. Drug Alcohol Depend. (2020) 213:108087. doi: 10.1016/j.drugalcdep.2020.108087, PMID: 32492601

[17] Brown DG Flanagan JC Jarnecke A Killeen TK Back SE. Ethnoracial differences in treatment-seeking veterans with substance use disorders and co-occurring PTSD: presenting characteristics and response to integrated exposure-based treatment. J Ethn Subst Abus. (2022) 21:1141–64. doi: 10.1080/15332640.2020.1836699, PMID: 33111647 PMC8079537

[18] Gasparyan A Navarro D Navarrete F Manzanares J. Pharmacological strategies for post-traumatic stress disorder (PTSD): from animal to clinical studies. Neuropharmacology. (2022) 218:109211. doi: 10.1016/j.neuropharm.2022.109211 35973598

[19] Lancaster CL Teeters JB Gros DF Back SE. Posttraumatic stress disorder: overview of evidence-based assessment and treatment. J Clin Med. (2016) 5:105. doi: 10.3390/jcm5110105, PMID: 27879650 PMC5126802

[20] Albott CS Lim KO Forbes MK Erbes C Tye SJ Grabowski JG. Efficacy, safety, and durability of repeated ketamine infusions for comorbid posttraumatic stress disorder and treatment-resistant depression. J Clin Psychiatry. (2018) 79:17462. doi: 10.4088/JCP.17m11634 29727073

[21] Jones JL Mateus CF Malcolm RJ Brady KT Back SE. Efficacy of ketamine in the treatment of substance use disorders: a systematic review. Front Psych. (2018) 9:277. doi: 10.3389/fpsyt.2018.00277, PMID: 30140240 PMC6094990

[22] Dakwar E Nunes EV Hart CL Foltin RW Mathew SJ Carpenter KM. A single ketamine infusion combined with mindfulness-based behavioral modification to treat cocaine dependence: a randomized clinical trial. Am J Psychiatr. (2019) 176:923–30. doi: 10.1176/appi.ajp.2019.18101123, PMID: 31230464

[23] Grabski M McAndrew A Lawn W Marsh B Raymen L Stevens T. Adjunctive ketamine with relapse prevention–based psychological therapy in the treatment of alcohol use disorder. Am J Psychiatr. (2022) 179:152–62. doi: 10.1176/appi.ajp.2021.21030277, PMID: 35012326

[24] Bogenschutz MP Forcehimes AA Pommy JA Wilcox CE Barbosa PC Strassman RJ. Psilocybin-assisted treatment for alcohol dependence: a proof-of-concept study. J Psychopharmacol. (2015) 29:289–99. doi: 10.1177/0269881114565144, PMID: 25586396

[25] Bogenschutz MP Ross S Bhatt S Baron T Forcehimes AA Laska E. Percentage of heavy drinking days following psilocybin-assisted psychotherapy vs placebo in the treatment of adult patients with alcohol use disorder: a randomized clinical trial. JAMA Psychiatry. (2022) 79:953–62. doi: 10.1001/jamapsychiatry.2022.2096, PMID: 36001306 PMC9403854

[26] Johnson MW Garcia-Romeu A Cosimano MP Griffiths RR. Pilot study of the 5-HT2AR agonist psilocybin in the treatment of tobacco addiction. J Psychopharmacol. (2014) 28:983–92. doi: 10.1177/0269881114548296, PMID: 25213996 PMC4286320

[27] Johnson MW Garcia-Romeu A Griffiths RR. Long-term follow-up of psilocybin-facilitated smoking cessation. Am J Drug Alcohol Abuse. (2017) 43:55–60. doi: 10.3109/00952990.2016.1170135, PMID: 27441452 PMC5641975

[28] Feder A Costi S Rutter SB Collins AB Govindarajulu U Jha MK. A randomized controlled trial of repeated ketamine administration for chronic posttraumatic stress disorder. Am J Psychiatr. (2021) 178:193–202. doi: 10.1176/appi.ajp.2020.20050596, PMID: 33397139

[29] Mitchell JM Bogenschutz M Lilienstein A Harrison C Kleiman S Parker-Guilbert K. MDMA-assisted therapy for severe PTSD: a randomized, double-blind, placebo-controlled phase 3 study. Nat Med. (2021) 27:1025–33. doi: 10.1038/s41591-021-01336-3, PMID: 33972795 PMC8205851

[30] Nicholas CR Wang JB Coker A Mitchell JM Klaire SS Yazar-Klosinski B. The effects of MDMA-assisted therapy on alcohol and substance use in a phase 3 trial for treatment of severe PTSD. Drug Alcohol Depend. (2022) 233:109356. doi: 10.1016/j.drugalcdep.2022.109356, PMID: 35286849 PMC9750500

[31] Sessa B Higbed L O’Brien S Durant C Sakal C Titheradge D. First study of safety and tolerability of 3, 4-methylenedioxymethamphetamine-assisted psychotherapy in patients with alcohol use disorder. J Psychopharmacol. (2021) 35:375–83. doi: 10.1177/0269881121991792, PMID: 33601929

[32] U.S. Census Bureau (2021) Quick facts. Available at: https://www.census.gov/quickfacts/fact/table/US/PST045219 (Accessed November 1, 2022).

[33] Michaels TI Purdon J Collins A Williams MT. Inclusion of people of color in psychedelic-assisted psychotherapy: a review of the literature. BMC Psychiatry. (2018) 18:245. doi: 10.1186/s12888-018-1824-6, PMID: 30064392 PMC6069717

[34] Ching TH Williams MT Wang JB Jerome L Yazar-Klosinski B Emerson A. MDMA-assisted therapy for posttraumatic stress disorder: a pooled analysis of ethnoracial differences in efficacy and safety from two phase 2 open-label lead-in trials and a phase 3 randomized, blinded placebo-controlled trial. J Psychopharmacol. (2022) 36:974–86. doi: 10.1177/02698811221104052, PMID: 35727042

[35] Williams MT Davis AK Xin Y Sepeda ND Grigas PC Sinnott S. People of color in North America report improvements in racial trauma and mental health symptoms following psychedelic experiences. Drugs Educ Prev Policy. (2021) 28:215–26. doi: 10.1080/09687637.2020.1854688, PMID: 34349358 PMC8330400

[36] George JR Michaels TI Sevelius J Williams MT. The psychedelic renaissance and the limitations of a white-dominant medical framework: a call for indigenous and ethnic minority inclusion. J Psychedelic Stud. (2019) 4:4–15. doi: 10.1556/2054.2019.015

[37] George S Duran N Norris K. A systematic review of barriers and facilitators to minority research participation among African Americans, Latinos, Asian Americans, and Pacific islanders. Am J Public Health. (2014) 104:e16–31. doi: 10.2105/AJPH.2013.301706, PMID: 24328648 PMC3935672

[38] Smith DT Faber SC Buchanan NT Foster D Green L. The need for psychedelic-assisted therapy in the black community and the burdens of its provision. Frontiers in Psychiatry. (2022) 12:774736. doi: 10.3389/fpsyt.2021.774736 35126196 PMC8811257

[39] Chan, Michelle Stephanie (2017). Coping with acculturative stress: MDMA usage among Asian American young adults in the electronic dance music scene. Pomona Senior Theses, 194.

