# Supplementary material for: Perspectives on the therapeutic potential of MDMA: A nation-wide exploratory survey among substance users
Source: Front Psychiatry. 2023 Apr 14;14:1096298. doi: 10.3389/fpsyt.2023.1096298 (PMC10140372; doi:10.3389/fpsyt.2023.1096298)
Supplement: Supplementary file 1 [file Data_Sheet_1.PDF]

Supplemental Figure 2: Opinions on Psychedelic Therapy Survey

Opinions On Psychedelic Therapy

Please complete the survey below.

Thank you!

What is your current age?

- ◯ 18 to 24
- ◯ 25 to 34
- ◯ 35 to 44
- ◯ 45 to 54
- ◯ 55+
- ◯ Prefer not to answer

What gender do you consider yourself?

- ◯ Male
- ◯ Female
- ◯ Transgender or non-binary
- ◯ Other or prefer not to answer

Region Map

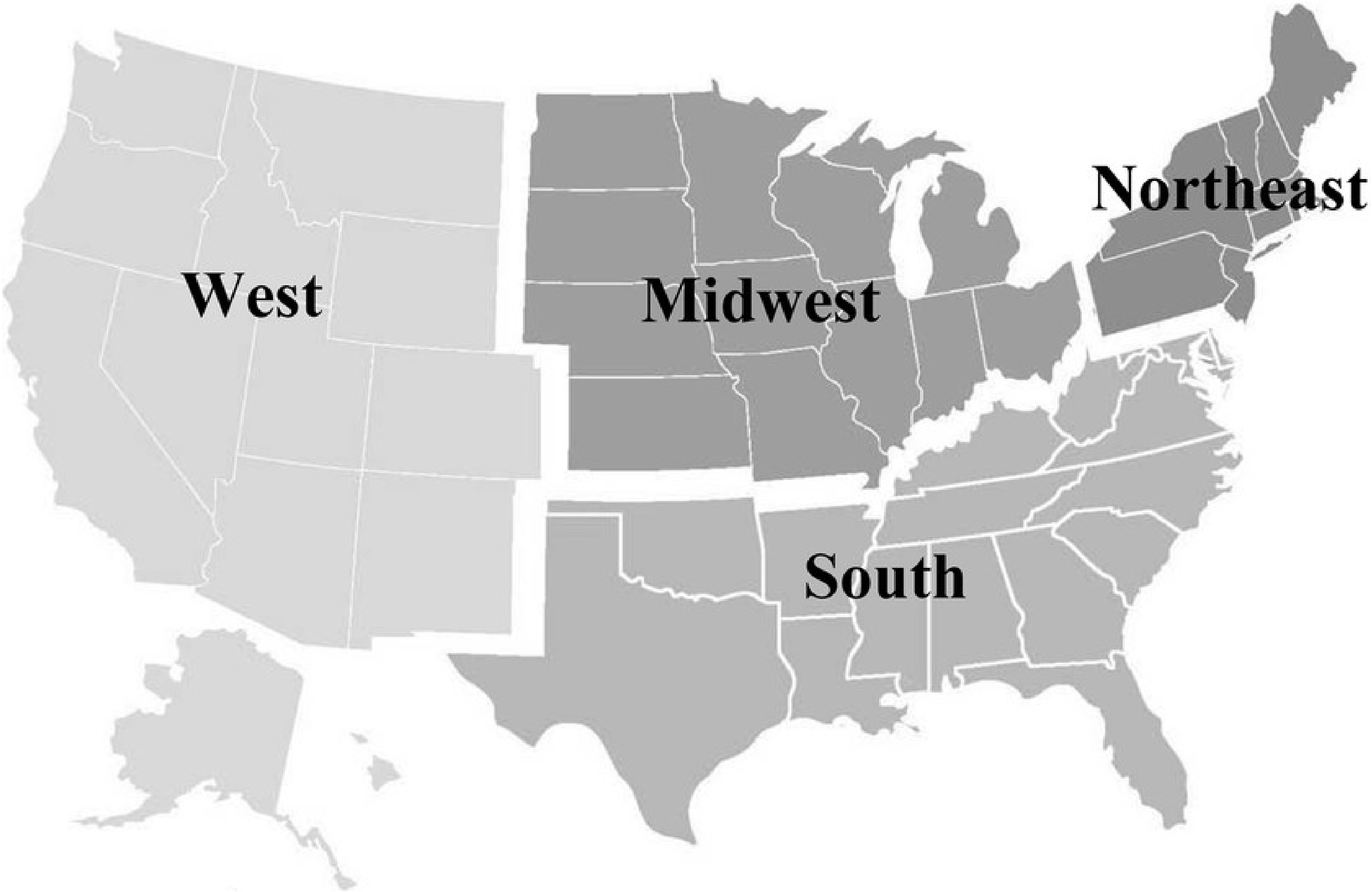

Based on the map above, what region do you live in?

- ◯ Northeast
- ◯ Midwest
- ◯ South
- ◯ West

What size is your community approximately?	◯ Large city<br>◯ Midsized city<br>◯ Small city<br>◯ Suburban<br>◯ Rural
What is the highest level of education that you have completed?	◯ No high school<br>◯ Some high school<br>◯ High school graduate or have GED<br>◯ Some college<br>◯ Completed two-year college degree<br>◯ Completed four year college degree<br>◯ Have had some graduate degree training<br>◯ Have completed graduate degree training
What is your relationship status?	◯ Married<br>◯ Separated<br>◯ Divorced<br>◯ Widowed<br>◯ Never married<br>◯ Domestic/civil partnership
Do you have children under the age of 18?	◯ Yes<br>◯ No
What race/ethnicity do you consider yourself?	☐ American Indian or Alaska Native<br>☐ Asian<br>☐ Black or African American<br>☐ Hispanic or Latino<br>☐ Native Hawaiian or Other Pacific Islander<br>☐ White<br>☐ Other or prefer not to say
Which of the following substances do you use on a regular basis (at least once per month)?<br><br>Please mark all that apply.	☐ Alcohol<br>☐ Tobacco<br>☐ Marijuana<br>☐ Cocaine<br>☐ Opiates<br>☐ Amphetamines<br>☐ Benzodiazepines<br>☐ Other drugs not listed above<br>☐ None of the above
If other, please list.	______________________________
Have you ever wanted to cut back or quit using any of those substances?	◯ Yes<br>◯ No
Have the use of any of these substances ever caused you trouble with work, with your family or friends, or legal trouble?	◯ Yes<br>◯ No

Have you ever previously tried any of the following psychedelic substances? Please mark all that apply:	☐ Psilocybin<br>☐ LSD<br>☐ Peyote<br>☐ Ibogaine<br>☐ Ayahuasca<br>☐ Ketamine<br>☐ Other<br>☐ None of the above
If other, please list.	______________________
Have you ever been in any form of treatment for alcohol or drug use?	○ Yes<br>○ No
Are you currently in any form of treatment for alcohol or drug use?	○ Yes<br>○ No
Have you ever received any mental health treatment?	○ Yes<br>○ No
Have you ever previously heard about the use of psychedelic-type substances (such as psilocybin, LSD, peyote, ibogaine, ayahuasca, and ketamine) being used in the treatment of addictions?	○ Yes<br>○ No

Psilocybin ("magic mushrooms") has been illegal in the United States for more than 40 years. However:

*** A recent study of tobacco smokers at Johns Hopkins showed after 2 to 3 psilocybin treatments, 2/3 (67%) of participants were able to quit smoking for more than one year. Participants had minimal side effects.**

***Another small study in heavy alcohol users showed that after 2 psilocybin treatments, participants were able to cut back on their number of heavy drinking days by half (50%). On average, participants were able to sustain this improvement throughout the six months of the study.**

Based on these preliminary findings, and anything else you may know:<br><br>Do you support or oppose similar medical trials with psilocybin being conducted in the future?	○ Strongly oppose<br>○ Oppose<br>○ Neutral or unsure<br>○ Support<br>○ Strongly support
Do you think psilocybin could be a beneficial treatment for people suffering with addictions?	○ Definitely not<br>○ Probably not<br>○ Neutral or unsure<br>○ Probably yes<br>○ Definitely yes

If psilocybin is proven to be safe and effective for the treatment of addictions, would you or would you not try this treatment if it was appropriate for you?	◯ Definitely not<br>◯ Probably not<br>◯ Neutral or unsure<br>◯ Probably would<br>◯ Definitely would

Ketamine, a medication used for the past forty years to reduce pain when undergoing surgeries, has also been studied for the treatment of addictions.

*** A previous study showed that after 3 treatments with ketamine in conjunction with therapy, 50% of study participants were able to be abstinent from heroin for over one year.**

*** A related study in heavy alcohol users found that after a session of ketamine in conjunction with psychotherapy, nearly 2/3 of study participants were able to maintain abstinence for over one year.**

*** Other studies in cocaine users have shown that motivation to quit increases following a ketamine treatment.**

Based on these preliminary findings, and anything else you may know:<br><br>Do you support or oppose similar medical trials with ketamine being conducted in the future?	◯ Strongly oppose<br>◯ Oppose<br>◯ Neutral or unsure<br>◯ Support<br>◯ Strongly support
Do you think ketamine could be a beneficial treatment for people suffering with addictions?	◯ Definitely not<br>◯ Probably not<br>◯ Neutral or unsure<br>◯ Probably yes<br>◯ Definitely yes
If ketamine is proven to be safe and effective for treatment after further trials, would you or would you not try this treatment if it was appropriate for you?	◯ Definitely not<br>◯ Probably not<br>◯ Neutral or unsure<br>◯ Probably would<br>◯ Definitely would

In 2016, the FDA approved MDMA (also known as ecstasy) for Phase 3 clinical trials as a treatment for post-traumatic stress disorder (PTSD), which is a common disorder that occurs with addictions. These studies are one of the final steps before possible approval as a prescription drug.

*** One previous study showed that with 3 doses of MDMA administered under a psychiatrist's guidance, the patients reported a 56% decrease in severity of PTSD symptoms on average.**

*** At the end of the study, 2/3 of the study participants (66%) no longer met the criteria for having PTSD.**

*** Improvements lasted more than a year after therapy.**

Based on these findings and what you may have known previously, do you support or oppose similar medical trials with MDMA being conducted in the future?	◯ Strongly oppose<br>◯ Oppose<br>◯ Neutral or unsure<br>◯ Support<br>◯ Strongly support
Do you think MDMA could or could not be a beneficial treatment for people suffering with PTSD?	◯ Definitely not<br>◯ Probably not<br>◯ Neutral or unsure<br>◯ Probably yes<br>◯ Definitely yes
If MDMA is proven to be safe and effective for treatment after further trials, would you or would you not try this treatment if it was appropriate for you?	◯ Definitely would<br>◯ Probably would<br>◯ Neutral or unsure<br>◯ Probably would not<br>◯ Definitely would not
Do you have any of the following concerns about using psychedelics (psilocybin, ketamine, MDMA, or other psychedelic medications) to reduce or stop using alcohol or other drugs?<br><br>Please mark all that apply.	☐ Fear of a bad trip<br>☐ Fear that you wouldn't enjoy using your substance of choice as much afterwards<br>☐ Fear that it would "change you"<br>☐ Fear that it would cause you to "go crazy" or "lose your mind"<br>☐ Fear that you would harm yourself or others while on a bad trip<br>☐ Fear that you would feel guilt during your trip<br>☐ Fear that you would lose your sense of self<br>☐ Fear that it would affect your employment<br>☐ Fear that your family, neighbors, or others in the community would have a negative opinion if they found out that you tried this type of treatment<br>☐ Have concerns other than those listed above<br>☐ Don't have any concerns
If other concerns, please list.	________________________________
Do any of the following concerns apply to you?	☐ Belief that the treatment would be too expensive<br>☐ Feeling like you are not ready to stop drinking alcohol or using drugs<br>☐ Feeling like life would be boring or not worth living if you stopped drinking alcohol or using drugs<br>☐ Fear that your family, neighbors, or others in the community would have a negative opinion if they found out that you have an addiction or trouble controlling your use of substances<br>☐ Don't have any of these concerns
Which of the following would you be INTERESTED IN trying as a treatment to reduce or quit using alcohol or drugs? Please mark all that apply.	☐ Psilocybin<br>☐ Ketamine<br>☐ MDMA<br>☐ Not interested in trying any of these
Which of the following would you be CONCERNED ABOUT trying as a treatment to reduce or quit using alcohol or drugs?<br><br>Please mark all that apply.	☐ Psilocybin<br>☐ Ketamine<br>☐ MDMA<br>☐ No concerns about trying any of these

Several other substances have been studied in other countries for the treatment of addictions with promising results.<br><br>Which of the following would you be INTERESTED IN trying, if it was available as a treatment in the United States?	☐ LSD<br>☐ Peyote<br>☐ Ibogaine<br>☐ Ayahuasca<br>☐ Not interested in trying any of these
Several other substances have been studied in other countries for the treatment of addictions with promising results.<br><br>Which of the following would you be CONCERNED about trying, if it was available as a treatment in the United States?	☐ LSD<br>☐ Peyote<br>☐ Ibogaine<br>☐ Ayahuasca<br>☐ No concerns about trying any of these
