# Supplementary figures and images for: Perspectives on the therapeutic potential of MDMA: A nation-wide exploratory survey among substance users
Source: Front Psychiatry. 2023 Apr 14;14:1096298. doi: 10.3389/fpsyt.2023.1096298 (PMC10140372; doi:10.3389/fpsyt.2023.1096298)

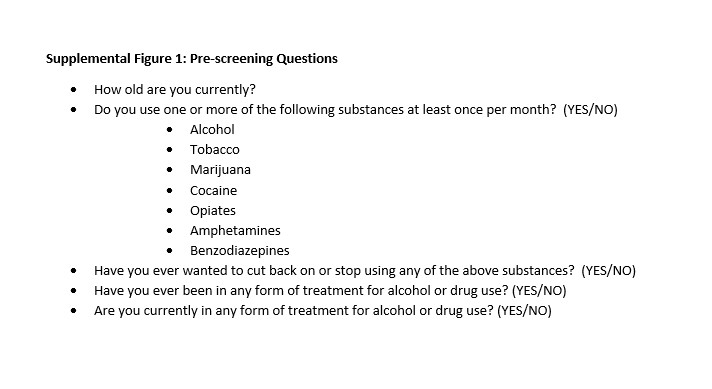

Supplement: Supplementary file 2 [file Image_1.JPEG]
